# Supplementary material for: Rtf1-dependent transcriptional pausing regulates cardiogenesis
Source: eLife. 2026 Jan 15;13:RP94524. doi: 10.7554/eLife.94524 (PMC12807453; doi:10.7554/eLife.94524)
Supplement: Figure 1—source data 1. [file elife-94524-fig1-data1.zip › Figure 1 Source Data 1.pdf]

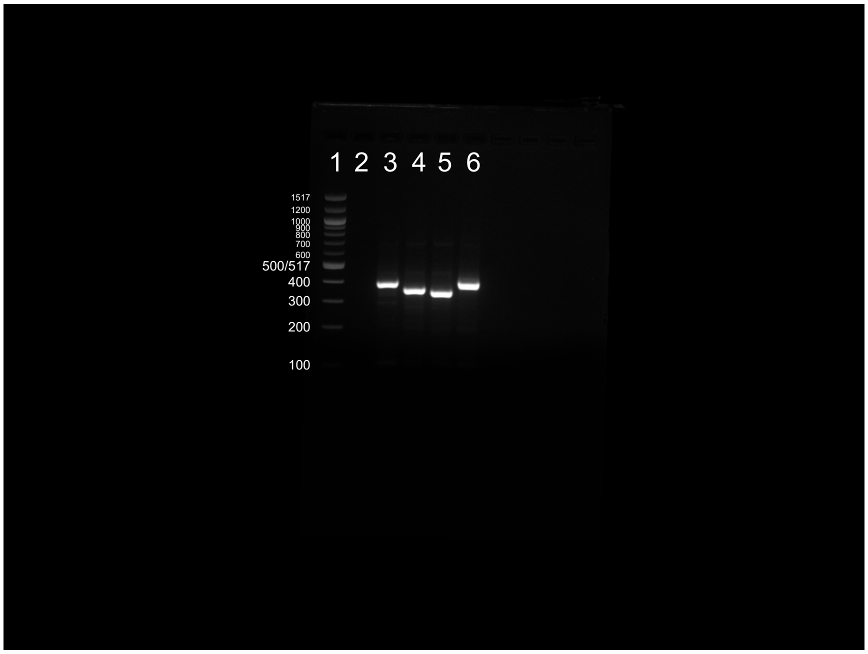

Lanes:

1: NEB 100 bp Ladder (bp labeled to the left)

2: Intentionally left empty

3: Product from rtf1-e3-F and rtf1-e3-R primer PCR on gDNA from wild type sibling of rtf1 mutant in Lane 4.

4: Product from rtf1-e3-F and rtf1-e3-R primer PCR on gDNA from rtf1 LA2678 mutant.

5: Product from rtf1-e3-F and rtf1-e3-R primer PCR on gDNA from rtf1 LA2679 mutant.

6: Product from rtf1-e3-F and rtf1-e3-R primer PCR on gDNA from wild type sibling of rtf1 mutant in Lane 5.
