# Supplementary figures and images for: Rtf1-dependent transcriptional pausing regulates cardiogenesis
Source: eLife. 2026 Jan 15;13:RP94524. doi: 10.7554/eLife.94524 (PMC12807453; doi:10.7554/eLife.94524)

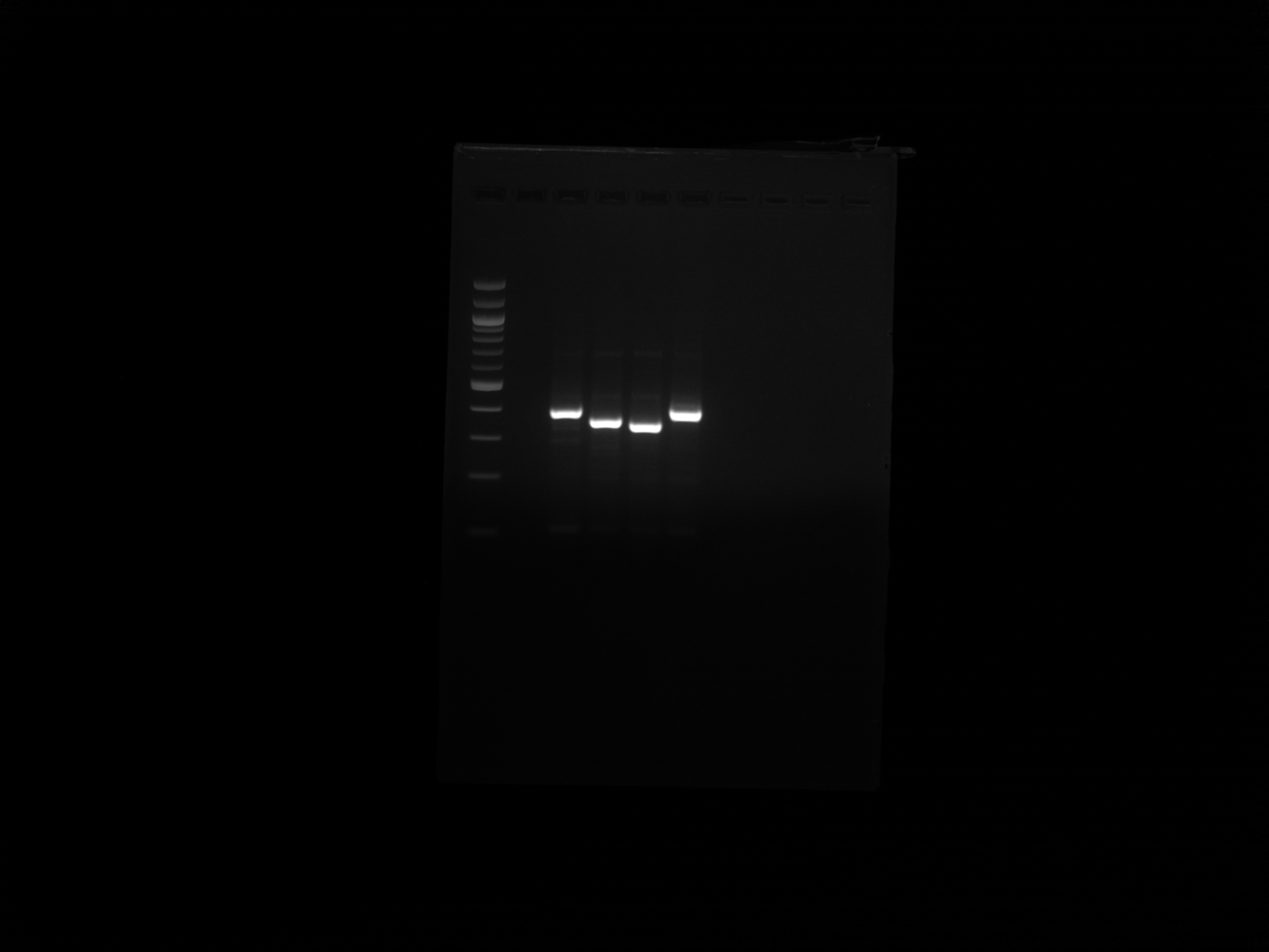

Supplement: Figure 1—source data 2. [file elife-94524-fig1-data2.zip › Figure 1 Source Data Agarose Gel.tif]

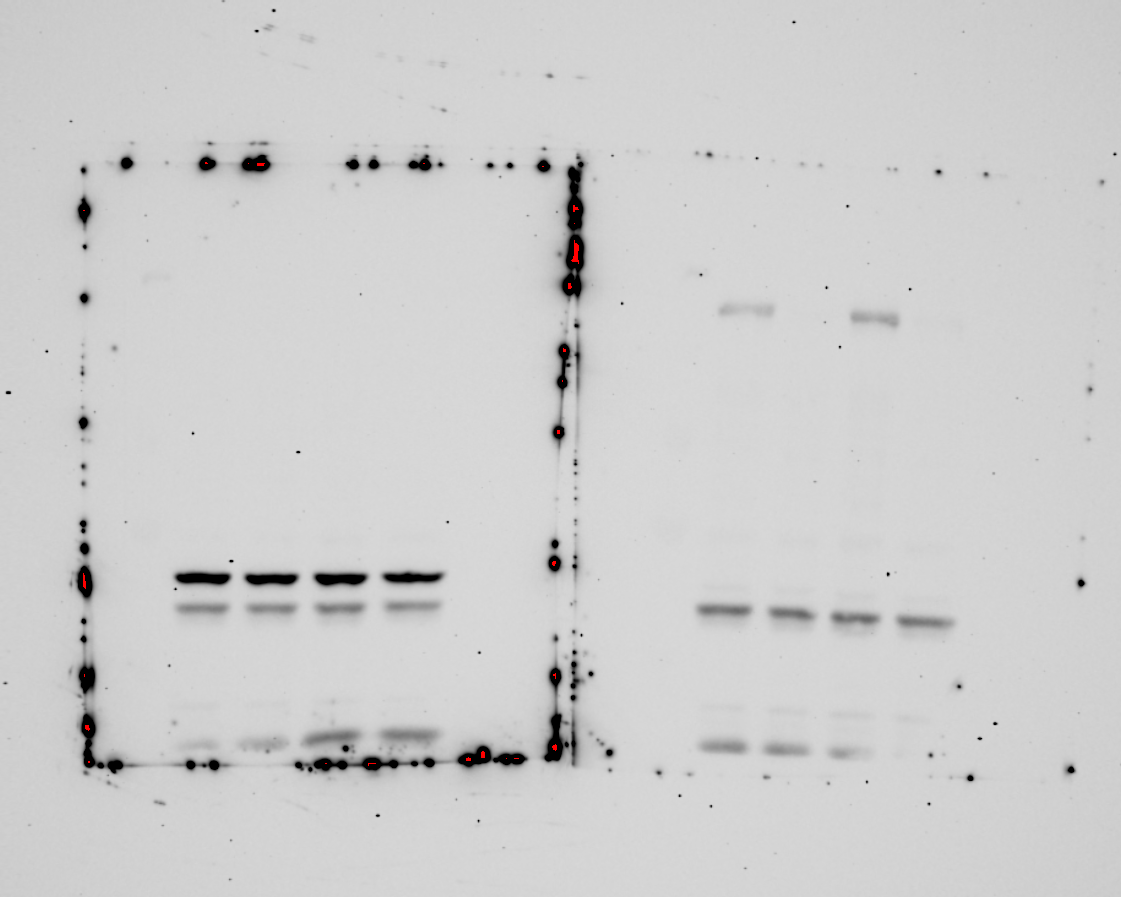

Supplement: Figure 1—source data 4. [file elife-94524-fig1-data4.zip › Figure 1 Source Data Western Blot Exposure 1.tif]

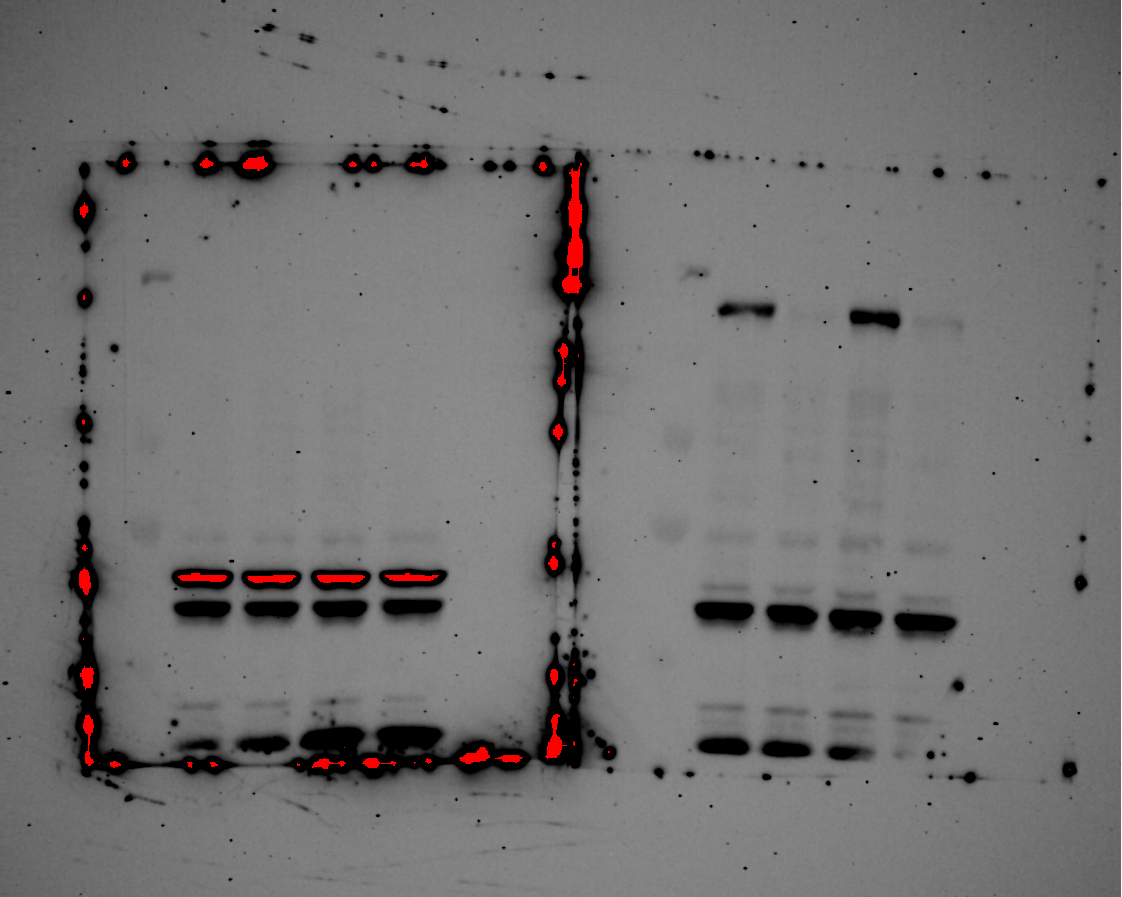

Supplement: Figure 1—source data 4. [file elife-94524-fig1-data4.zip › Figure 1 Source Data Western Blot Exposure 2.tif]

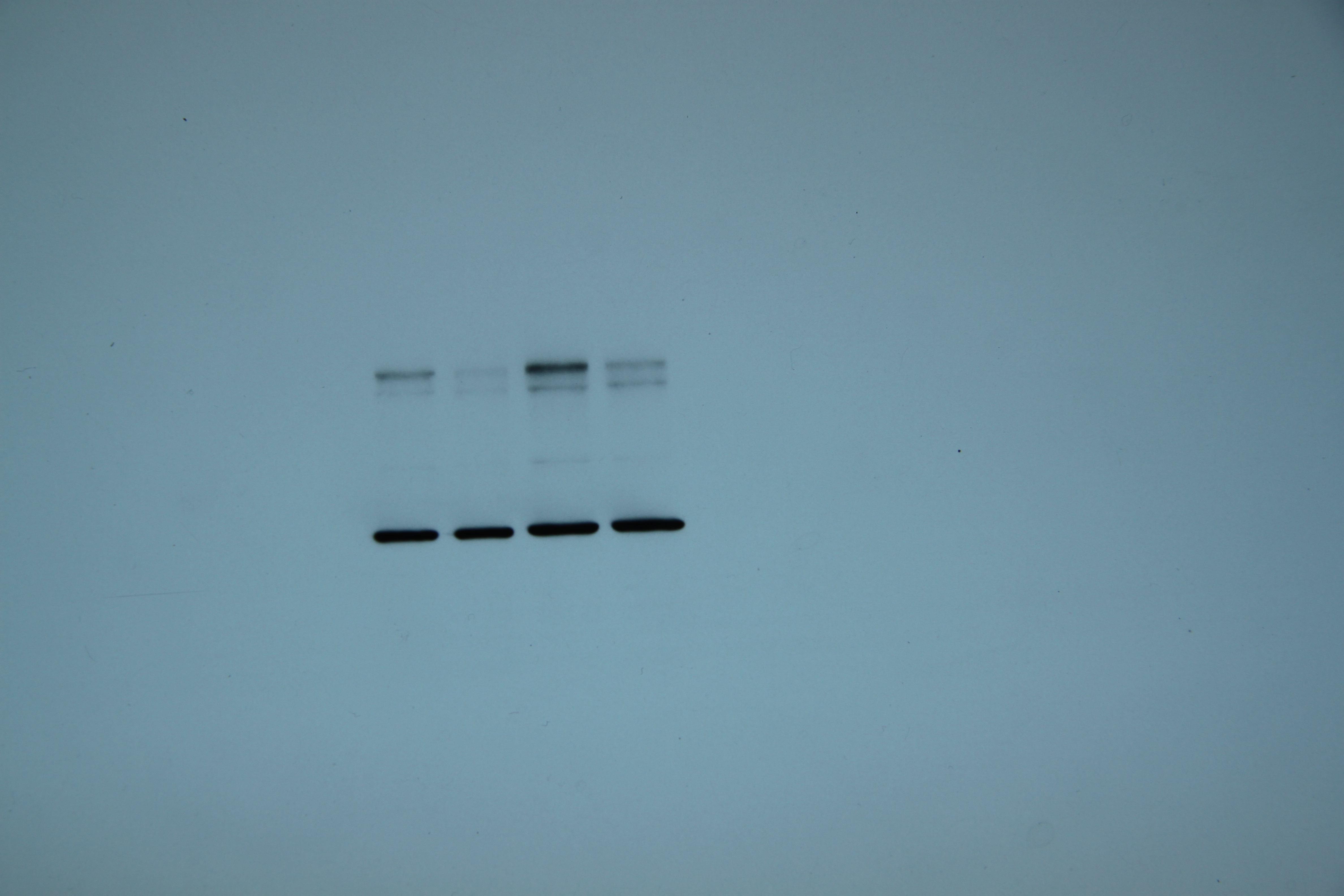

Supplement: Figure 4—source data 2. [file elife-94524-fig4-data2.zip › Figure 4 Source Data Western Blot.JPG]
