# Supplementary material for: Rtf1-dependent transcriptional pausing regulates cardiogenesis
Source: eLife. 2026 Jan 15;13:RP94524. doi: 10.7554/eLife.94524 (PMC12807453; doi:10.7554/eLife.94524)
Supplement: Figure 1—source data 3. [file elife-94524-fig1-data3.zip › Figure 1 Source Data 3.pdf]

40.256 second exposure

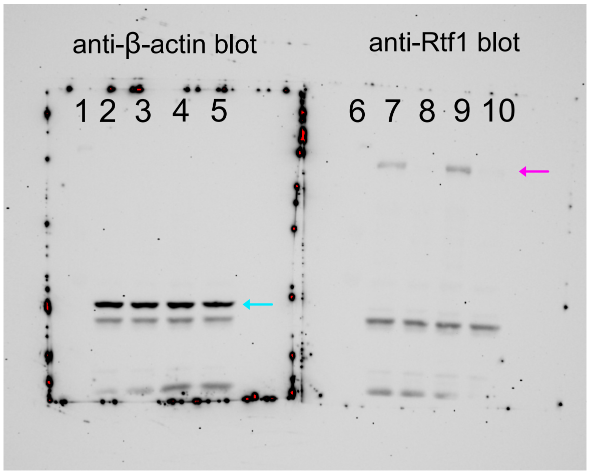


267.176 second exposure

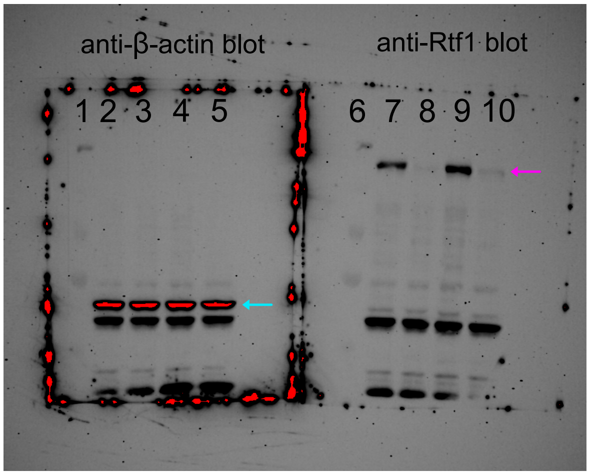


Cyan arrow indicates β-actin protein.

Magenta arrow indicates Rtf1 protein.

Lanes:

1: Spectra Multicolor Broad Range Protein Ladder (bands too light to accurately label molecular weights)

2: Protein lysate of wildtype siblings of rtf1 mutants in Lane 3.

3: Protein lysate of rtf1 LA2678 mutants.

4: Protein lysate of wildtype siblings of rtf1 mutants in Lane 5.

5: Protein lysate of rtf1 LA2679 mutants.

6: Spectra Multicolor Broad Range Protein Ladder (bands too light to accurately label molecular weights)

7: Protein lysate of wildtype siblings of rtf1 mutants in Lane 8.

8: Protein lysate of rtf1 LA2678 mutants.

9: Protein lysate of wildtype siblings of rtf1 mutants in Lane 10.

10: Protein lysate of rtf1 LA2679 mutants.
