# Supplementary material for: Rtf1-dependent transcriptional pausing regulates cardiogenesis
Source: eLife. 2026 Jan 15;13:RP94524. doi: 10.7554/eLife.94524 (PMC12807453; doi:10.7554/eLife.94524)
Supplement: Figure 4—source data 1. [file elife-94524-fig4-data1.zip › Figure 4 Source Data 1.pdf]

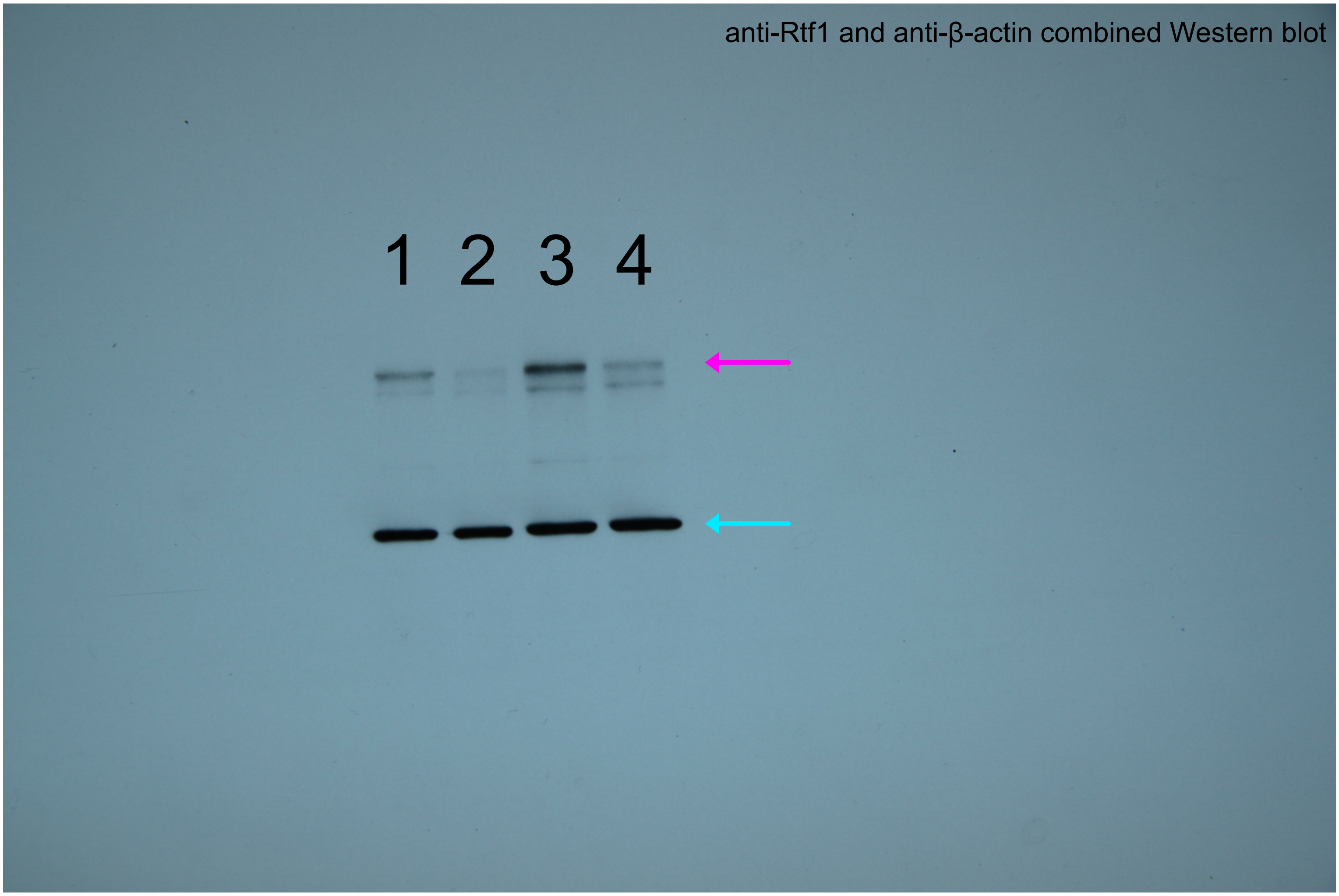

Cyan arrow indicates β-actin protein.

Magenta arrow indicates Rtf1 protein.

Lanes:

1: Protein lysate from Non-target shRNA-treated mESCs

2: Protein lysate from Rtf1 shRNA-treated mESCs

3: Protein lysate from Non-target shRNA-treated mESCs

4: Protein lysate from Rtf1 shRNA-treated mESCs
